# Sternal fractures and thoracic injury: an analysis of 288 sternal fractures attending a major trauma centre

**DOI:** 10.1007/s00590-023-03479-0

**Published:** 2023-02-03

**Authors:** Junaid Aamir, Bolutife Alade, Robyn Caldwell, James Chapman, Sohan Shah, Dileep Karthikappallil, Luke Williams, Lyndon Mason

**Affiliations:** 1 Aintree University Hospital, Liverpool, UK; 2 University of Liverpool, Liverpool, UK; 3 Department of Trauma and Orthopaedics, Aintree University Hospital, Liverpool, UK

**Keywords:** Sternal fracture, Major trauma, Thoracic injury

## Abstract

**Introduction:**

Sternal fractures (SF) are uncommon injuries usually associated with a significant mechanism of injury. Concomitant injury is likely, and a risk of mortality is substantial.

**Aim:**

Our aim in this study was to identify the risk factors for mortality in patients who had sustained sternal fractures.

**Methods:**

We conducted a single centre retrospective review of the trust’s Trauma Audit and Research Network Database, from May 2014 to July 2021. Our inclusion criteria were any patients who had sustained a sternal fracture. The regions of injury were defined using the Abbreviated Injury Score. Pearson Chi-Squared, Fisher Exact tests and multivariate regression analyses were performed using IBM SPSS.

**Results:**

A total of 249 patients were identified to have sustained a SF. There were 19 patients (7.63%) who had died. The most common concomitant injuries with SF were Rib fractures (56%), Lung Contusions (31.15%) and Haemothorax (21.88%). There was a significant increase in age (59.93 vs 70.06, *p* = .037) and admission troponin (36.34 vs. 100.50, *p* = .003) in those who died. There was a significantly lower GCS in those who died (10.05 vs. 14.01, *p* < .001). On multi regression analysis, bilateral rib injury (*p* = 0.037, OR 1.104) was the only nominal variable which showed significance in mortality.

**Conclusion:**

Sternal Fractures are uncommon but serious injuries. Our review has identified that bilateral rib injuries, increase in age, low GCS, and high admission troponin in the context of SF, were associated with mortality.

## Introduction

Post traumatic sternal fractures (SF) are uncommon injuries, occurring in approximately 3–8% of blunt traumas [1]. Such injuries are typically consequential of high-impact forces to the thorax commonly associated with motor vehicle collisions (MVC) [2]. The incidence of SF has reportedly increased with mandatory seatbelt laws; presenting alone in isolated sternal fractures (ISF) or as part of a constellation injuries that occur following MVC, termed “seatbelt syndrome” [3–6]. A fracture of the sternum is referred to in literature as either primarily transverse or oblique.

Classically, SF injuries in isolation are often perceived as relatively benign. Cause for concern derives from associated injuries particularly to the chest cavity and internal organs [7]. Concomitant injury can include damage to the internal thoracic organs, thoracic cage (e.g., rib fractures, flail chest vertebral fractures), clavicle or scapular fractures [8]. A sharp rise in morbidity and mortality is displayed in SF associated with concomitant injuries ranging from 4 to 45% [9]. Injuries to the brain and abdominal cavity are also commonly reported [10]. Notably, the risk of blunt cardiac injury (BCI) is theorised to increase morbidity and mortality in SF. When SF is comminuted or depressed, as can occur with steering wheel impact, the heart and major blood vessels are prone to trauma [11]. BCI can present in a varied range of forms, including but not limited to new onset arrhythmia, recurrent tachycardia, murmurs, acute heart failure, and coronary artery occlusion preceding myocardial infarction [12]. Overall, research addressing BCI in patients with various forms of SF, as well as in cohorts of individuals with pre-existing cardiac problems, are few. It is estimated that 15–75% of patients sustaining blunt chest wall trauma may have sustained a concomitant BCI [13].

Currently, there is a lack of congruence across literature on how best to approach the assessment and management of SFs and their associated injuries. This is particularly true of BCI which has been established to cause an increase in mortality and morbidity. As such, in this study we aim to retrospectively review patients attending a level 1 major trauma centre to identify risk factors related to mortality in patients who had sustained a SF. Secondary outcome included assessing the prevalence of associated chest injuries in this cohort of patients.

## Material and methods

All patients from Cheshire and Merseyside Major Trauma Network who were admitted to Aintree University Hospital Major Trauma centre between May 2014 and July 2021 were included for review. Data was obtained from our institutions Trauma and Audit Research Network (TARN) database. Patients with SF documented in their injury summary were included in this study. We included patients with all mechanisms of injury as well as patients with isolated SF and SF with multisystem trauma.

Patient Information was retrospectively collected from Electronic Document Management System (EDMS) regarding patient demographics, mechanism of injury, associated injuries, Injury Severity Score (ISS), mortality, and method of diagnosis. We also recorded investigations performed on these patients such as cardiac biomarkers (troponin), ECG and Echocardiogram. Additionally, we recorded presence of associated thoracic and mediastinal injuries; SF with other thoracic injuries (SFOTI) such as cardiac injury, large vessel injury, pneumo-mediastinum, haemo-mediastinum, rib fractures, pneumothorax, haemothorax, and lung contusion were also recorded.

The diagnosis of sternal fracture was confirmed by analysing plain radiographs, and CT scans from our Picture Archiving and Communication System (PACS). For the purposes of this study, we recorded the type of SF based on their anatomical location: manubrium, superior body, mid body, inferior body, and xiphisternum. If more than one fracture was observed, we recorded both accordingly including whether they were displaced or undisplaced fractures. Displacement was categorised a > 2 mm of displacement in the SF.

Statistical analysis was performed using SPSS 26 (IBM Corp, USA). Comparisons were made between group characteristics. Chi-squared and Fisher’s exact tests were used for categorical variables and independent samples *t*-tests and Mann–Whitney U test for variable means. Uni- and multivariate analyses were performed using univariate and multivariate logistic regression analysis to identify factors involved in mortality. Any factor which achieved significance on univariate analysis underwent further multivariant regression analysis. Significance was given to variables that reached *p* < 0.05.

## Results

The mean age for SF patients was 61 years (95% CI, 17.6, 101.4), usually presented with an average ISS of 22.5 (95% CI, 4, 75) and average GCS of 14(95% CI 3, 15). The most common region of the body that sustained the most severe injury was the chest. (Table 1) One hundred percent of patients had their SF diagnosed with CT scan. We have identified that Motor Vehicle Accident (MVA) is the most common mechanism of injury in SF patients, occurring in almost 48% of cases of SF (Table 2). There was no significant difference in injury severity scores (ISS) between MVA patients and those of another mechanism of trauma (23.98 ± 14.32 vs. 21.09 ± 13.0, *p* = 0.07).

**Table 1:** Descriptive characteristics of SF patients

Characteristic	Result	Notes
Age, Mean years (± SD)	60.7 (± 20.10)	*N* = 288
Female, *n* (%)	122 (42.36%)	*N* = 288
Male, *n* (%)	166 (57.64%)	*N* = 288
Injury Severity Score, Mean (± SD)	22.49 (± 13.77)	*N* = 288
Glasgow Come Scale, Mean (± SD)	13.64 (± 3.24)	*N* = 287
NICE Head injury criteria met, *n* (%)	26 (9.03%)	*N* = 288
Attending with Systolic BP < 110 mmHg, *n* (%)	72 (25%)	*N* = 288
Most Severe injured Body Region–Abdomen, *n* (%)	4 (1.39%)	*N* = 288
Most Severe injured Body Region–Chest, *n* (%)	147 (51.04%)	*N* = 288
Most Severe injured Body Region–Head, *n* (%)	3 (11.81%)	*N* = 288
Most Severe injured Body Region–Limbs, *n* (%)	17 (5.90%)	*N* = 288
Most Severe injured Body Region–Spine, *n* (%)	20 (6.94%)	*N* = 288
Most Severe injured Body Region–Multiple, *n* (%)	66 (22.92%)	*N* = 288
Mortality at 30 days, *n* (%)	25 (8.69%)	*N* = 288

**Table 2:** Mechanism of Injury of SF patients

Mechanism, *n* (%)	*N* = 288
Blow(s) without weapon	6 (2.08%)
Crush	1 (0.35%)
Fall less than 2 m	43 (14.93%)
Fall more than 2 m	99 (34.38%)
Shooting	1 (0.35%)
Motor Vehicle Accident	138 (47.92%)

From our review, 19 patients (7.63%) had died within 30 days of sustaining a SF. Assessing the characteristics of SF pattern and associated injury we can see that in SF patients, midbody fractures were the most common, with 34% of SF patients being affected. Alongside this, the most common associated thoracic injuries were rib fractures (56.6%), followed by lung contusions (31.15%) and haemothorax (21.88%). (Table 3) When assessing the cohort of patients who had died, several significant findings were obtained. We found that compared to the rest of the SF cohort, patients who died had a higher average age (59.93 vs. 70.06, *p* = 0.037), additionally, admission troponin levels were significantly higher (36.34 vs. 100.50, *p* = 0.003). Admission GCS was also identified to be significantly lower in those patients that had died compared to those who survived (10.05 vs. 14.01, *p* < 0.001). When assessing nominal variables of associated thoracic injury, a multi-regression analysis identified that bilateral rib fractures was significantly associated with mortality (*p* = 0.037, OR 1.104) (Table 4 and 5).

**Table 3:** SF Characteristics: Location of SF and frequencies of other thoracic injuries

Characteristic, n (%)	Result	Notes
Manubrium SF	54 (18.75%)	*N* = 250
Superior Body SF	65 (22.57%)	*N* = 250
Midbody SF	98 (34.03%)	*N* = 250
Inferior Body SF	30 (10.42%)	*N* = 249
Xiphisternum SF	5 (1.74%)	*N* = 250
Displaced SF	80 (27.78%)	*N* = 251
Pneumothorax	77 (26.74%)	*N* = 252
Haemothorax	63 (21.88%)	*N* = 252
Rib fractures	163 (56.60%)	*N* = 252
Flail Chest	60 (20.80%)	*N* = 252
Lung Contusion	107 (37.15%)	*N* = 252
Cardiac Tamponade	2 (0.69%)	*N* = 252
Cardiac Injury	5 (1.74%)	*N* = 252
Large Vessel Injury	2 (0.69%)	*N* = 252
Pneumomediastinum	30 (10.42%)	*N* = 252
Haemomediastinum	39 (13.54%)	*N* = 252

**Table 4:** Results of Multi-regression Analysis

	B	S.E	Wald	df	Sig	Exp (B)
*Nominal Variables in the Equation*						
Bilateral Rib Fracture	1.104	0.53	4.341	1	0.037	3.017
Flail	0.202	0.556	0.132	1	0.716	1.224
Lung Contusion	0.99	0.538	3.39	1	0.066	2.692
*Ordinal Variables in the Equation*						
Age	0.08	0.022	13.74	1	0	1.083
ISS	0.034	0.023	2.228	1	0.135	1.035
GCS	−0.339	0.083	16.689	1	0	0.713

Further analysis of the entire cohort identified that there was no significant association between rib fractures and intubation (*p* = 0.11). Additionally, there was no significant difference found in ISS within the rib fracture group compared to patients without rib fractures (17.97 ± 14.0 vs. 24.94 ± 13.0, *p* = 0.84).

We also found that displaced SF was present in approximately 28% of cases (Table 3). Assessing the cardiac testing and monitoring whilst in hospital, 67% of patients underwent admission Troponin-T testing (*n* = 194). Of those, 54% underwent repeat Troponin-T testing (*n* = 156). Variable data was obtained on further cardiac assessment. 173 patients had evidence of ECG assessment and of those, approximately 3% had an abnormal ECG. Continuous cardiac monitoring was only utilised in 5% of cases (*n* = 249) (Table 2).

Through the analysis, the patients were split into displaced SF and undisplaced SF to determine whether this was a contributing factor to more severe injury in the thorax. Troponin-T levels were compared between patients with displaced and undisplaced SFs. When comparing the change in first and second troponin levels between displaced and undisplaced SFs, no significant difference was seen (First Troponin *p* = 0.106, Second Troponin *p* = 0.175). Additionally, there was no significant change in Troponin-T values within the whole cohort when comparing first and second test values (*p* = 0.875). When analysing the displaced SF group further, no significant difference was found between ISS and length of stay (*p* = 0.613 and *p* = 0.99, respectively) (Tables 5, 6).

**Table 5:** Summary of cardiac investigations performed on SF patients

Investigation *n*, (%)	Result	Notes
Admission Troponin performed	194 (67.36%)	*N* = 242
Second Troponin test performed	156 (54.17%)	*N* = 233
ECG performed	158 (54.86%)	*N* = 173
Acute Abnormality on ECG	8 (2.78%)	*N* = 173
Echocardiogram Performed	20 (6.94%)	*N* = 243
Acute Abnormality on Echocardiogram	4 (1.39%)	*N* = 22
Cardiac Monitoring Utilised	15 (5.21%)	*N* = 249

**Table 6:** Assessment of Tropnin-T levels compared to displaced and undisplaced sternal fractures

Troponin Levels		Undisplaced SF		Displaced SF	
		First Troponin	Second Troponin	First Troponin	Second Troponin
Mean		40.83	50.57	40.93	51.68
95% CI	Lower Bound	25.2	28.78	24.96	27.25
	Upper Bound	56.46	72.35	56.9	76.12
Std. Deviation		78.375	109.213	60.192	92.099

Change in Troponin Undisplaced vs. Displaced SF	*p* value	Change in Troponin (1^st^ Troponin vs. 2^nd^ Tropnin)	*p* value
1st Trop in undisplaced vs displaced	0.106	Whole cohort	0.875
2nd trop in undisplaced vs displaced	0.175	Undisplaced SF Group	0.53
		Displaced SF Group	0.33

## Discussion

Over a 7-year period, 288 patients attended our Major Trauma Centre with SFs. Our study displayed a 30-day mortality rate of approximately 8%. The group of patients that had died were of a higher age, had higher initial troponin and lower admission GCS on average compared to patients who had survived with SFs. Bilateral rib injury was the sole associated injury identified on multi-regression analysis which was significantly associated with mortality in patients with a SF. Analysis of the entire cohort identified that displacement of SF did not prove to be a significant prognostic indicator for mortality.

Sternal fractures were commonly associated with rib fractures (56%), lung contusions (31.15%) and haemothorax (21.88%). Evidence around associated injuries with SFs appears to be in line with our findings. Al-Thani et al*.* [14] assessed the presence of rib fractures in patients attending with SF and identified that those with rib fractures were generally older (40.1 years ± 13.6 vs. 37.8 years ± 14.5), more likely to be intubated (33% vs 19%) and have higher chest abbreviated injury scales (AIS) (AIS 2.8 ± 0.6 vs. 2.2 ± 0.5). Concomitant injuries alongside rib fractures such as haemothorax and lung contusions were more evident in their analysis of SF patients. [14] This evidence is also supported by Yakar et al. [15] who identified that MVA was the most common mechanism of injury in their SF patients (66.4%, *N* = 128) and the most common thoracic pathology accompanying SFs was rib fractures (36%, *N* = 128). Despite the evidence displaying the prevalence of rib fractures and its associated risk in the context of SF, there is very little evidence to review regarding bilateral rib injury.

The mortality of SFs admitted was low at 8%, 30 days post injury. Again, this remains in line with other evidence to suggest that although injury severity can be high, mortality remains low [14, 16, 17]. Assessments performed by Simsek et al. highlighted a mortality rate of 11.1% in 115 patients with SF where mortality rates were significantly higher in comminuted SFs (*p* = 0.045). As we identified patients who had died attended with significantly lower GCS, this is supported in the literature as reviews have suggested that injuries to the head may be present in up to 48% of cases of SF [4, 18].

Our patients attended with a mean ISS of 22.49 and 25% of patients with SF attended with systolic BP of < 110 mmHg. The chest was the most severe injured body region in 51% of patients. This highlights clearly that SF is often associated with severe injuries, particularly to the chest which can compromise haemodynamic stability. Ishida et al. displayed evidence to suggest that hypotension on arrival to emergency department was an independent risk factor associated with blunt cardiac injury (adjusted odds ratio (AOR) 4.536, 95% confidence interval (CI) 3.802–5.412). [9] In our study, MVA was the most common mechanism of injury in SF patients (48%). Several similar studies have estimated that MVA contributes to approximately 60–90% of sternal fractures [16, 17]. Additionally, the most fractured region of the sternum was the midbody (34%). This is in keeping with the evidence to suggesting the MVA association with SF is due to sudden decelerating forces acting on the chest as it collides directly with safety restraints or the steering wheel [19, 20].

Several diagnostic tests such as ECG, troponin and echocardiogram have been suggested in the evaluation of patients with suspected BCI [21, 22] Sixty-seven percent of SF patients underwent troponin enzyme testing on admission and approximately 55% had ECGs performed. Echocardiogram was the least commonly used investigation with around 7% of patients undergoing testing. Even with these low numbers of testing, abnormalities in ECG and Echo were only found in 3% and 1% of cases respectively. It is important to highlight that this low frequency is most likely attributed to incomplete medical records for SF patients. Fokin et al. found from their review of 380 SF patients that BCI patients underwent all three tests of ECG, Echo and Cardiac Enzyme assessment more often than those patients with SF and no BCI (90% vs. 36%, *p* < 0.001). [13] Electrocardiogram was previously thought to detect between 40 to 80% of myocardial injuries in patients with blunt chest trauma, however despite many reports ECG alone is considered insufficient in the evaluation of patients with suspected BCI [23]. Cardiac-specific Troponins are sensitive and specific (91%) markers of myocardial injury and are released from myocardial cell membrane following a severe injury or ischaemia [21, 22, 24].

## Limitations

The retrospective nature of the study with resulted in gaps within analysis, particularly pertaining to cardiac investigations and monitoring of SF patients. Outcomes after 30 days were not assessed due to inability to obtain enough follow up data. Additionally,

Cardiac events after blunt chest trauma in patients without SF were not analysed. Where possible, datasets were utilised with minimal areas of incomplete data.

## Conclusion

Overall, our retrospective review identified that SFs are, uncommon but serious injuries. We have highlighted that SF patients who attended with a higher age, high admission troponin and lower GCS are at risk of more severe injury and mortality. Additionally, bilateral rib fractures were a significantly associated with mortality.

## Declarations

### Human and animal participants

All human studies have been approved by the appropriate ethics committee and have therefore been performed in accordance with the ethical standards laid down in the 1964 Declaration of Helsinki and its later amendments. National laws have been adhered to.
